# The early use of inhaled nitric oxide in premature infants requiring respiratory support

**DOI:** 10.1080/07853890.2023.2266633

**Published:** 2023-12-11

**Authors:** Ahmed Osman

**Affiliations:** The Ohio State University College of Medicine and Nationwide Children’s Hospital, Columbus, OH, USA

**Keywords:** Premature infants, extreme prematurity, inhaled nitric oxide, respiratory distress syndrome, hypoxic respiratory failure, persistent pulmonary hypertension of the newborn, neonatal intensive care

## Abstract

**Background:** Earlier studies on the use of inhaled nitric oxide (iNO) for premature infants born at <34 weeks of gestation requiring respiratory support did not provide conclusive evidence of benefit. National guidelines generally discouraged the use in this population. More recent national guidelines endorsed the use of iNO in premature infants with hypoxic respiratory failure (HRF) associated with persistent pulmonary hypertension of the newborn (PPHN).

**Recent Studies:** Two recently published observational studies evaluated the effect of administering iNO on oxygenation in the first week of life. These studies compared premature infants born at the gestational age (GA) of <34 weeks with HRF associated with PPHN to term and late preterm infants born at the GA of ≥34 weeks who received iNO. Both studies showed a similar effect of iNO on oxygenation in the two infant cohorts. The response rate in the premature infant cohort was 59% in the first study and 90% in the second. The mean response time was 9.2 h and 10.3 h, and the mean duration of therapy was 3.5 days and 8.2 days, respectively.

**Conclusion: **The results of these studies support a trial of iNO in premature infants with persistent hypoxia despite optimum respiratory support. Obtaining a timely echocardiogram to exclude cardiac diseases and diagnose PPHN is logistically challenging for many clinicians, thus, a clinical diagnosis of PPHN might have to be made in these situations. Questions remain regarding the optimum dose of iNO and the duration of the initial iNO trial in these patients.

**KEY MESSAGES**
- In the most recently published studies, the improvement of oxygenation in iNO-treated infants born at <34 weeks of gestation with HRF and PPHN physiology was as effective as in infants born ≥34 weeks.
- These studies provide evidence supporting a trial of iNO in the subpopulation of premature infants with HRF associated with PPHN.

## Introduction

Nitric oxide is a gas that occurs naturally and facilitates the adaptation of pulmonary circulation to extra-uterine life. Nitric oxide also has anti-inflammatory and modulating properties [1]. Randomized controlled clinical trials showed that inhaled nitric oxide (iNO) decreased the need for extracorporeal membrane oxygenation in term and late-preterm infants with persistent pulmonary hypertension of the newborn (PPHN) [2–5]. The United States Food and Drug Administration (FDA) approved the use of the drug in term and preterm infants born at >34 weeks of gestation with PPHN.

## Discussion

### Early studies on the use of iNO for preterm infants

Historically, many studies evaluated the use of iNO in preterm infants for various indications and outcomes. These include the use of iNO in preterm infants with hypoxic respiratory failure (HRF); for the prevention of bronchopulmonary dysplasia (BPD); and the effect of iNO on mortality and neurodevelopmental outcome [6–10]. Despite the lack of evidence for a clear benefit of iNO in this population, clinical use increased significantly. Thus, the National Institutes of Health (NIH) held a development conference to address the increasing use of iNO in premature infants and to assess the available evidence to provide a consensus statement for clinicians [11]. The NIH proposed to answer and make recommendations on the following effects of iNO in premature infants: mortality and BPD morbidity; the short-term risk; and the long-term respiratory and neurodevelopmental outcomes. The totality of evidence from the literature search and meta-analysis did not support a positive effect of iNO on the rate of mortality or BPD at 36 weeks postmenstrual age (PMA). Additionally, there was no evidence for improved neurodevelopmental outcome. However, some of the randomized controlled trials (RCTs) showed a significant reduction in the composite outcome of death or BPD at 36 weeks PMA [12]. The conclusion of NIH meeting was that the use of iNO in early routine, early rescue or later rescue regimens in the care of premature infants ≤34 weeks of gestation was not supported by the available evidence [11]. However, the meeting suggested that some clinical situations in which iNO may have benefits, including pulmonary hypertension, have been inadequately studied. The meeting’s recommendation left the clinical use in these situations at the discretion of treating clinicians [11].

### Evolution of consensus guidelines and clinical use

American Academy of Pediatrics (AAP) published a statement on the use of iNO in premature infants in 2014. The AAP statement was aligned with the NIH conference conclusion stating that neither rescue nor routine use of iNO improves survival in preterm infants with respiratory distress [13]. The statement also recommended against the use of iNO to prevent or ameliorate morbidities including BPD [13]. The guideline mentioned that iNO appears to be safe based on limited data [13]. The Cochrane Neonatal Group conducted a systematic review of studies evaluating the use of iNO as rescue therapy in very ill premature infants and the early routine use of iNO in premature infants with respiratory distress. They concluded that neither routine nor rescue use was associated with improved outcome [14].

Despite these recommendations, the clinical use of iNO in premature infants continues and has increased with noticeable variations between medical centres [15,16]. Three-quarters of centres with iNO administration capabilities in the Vermont Oxford Network (VON) treated premature infants born at <29 weeks with iNO, with an average of 8% of these infants having received iNO [17]. Many medical centres initiated iNO stewardship programs to optimize its use in premature infants [18–20].

In 2015, the American Heart Association and American Thoracic Society jointly published the paediatric pulmonary hypertension guidelines recommending the use of iNO in premature infants for the indication of pulmonary hypertension [21]. The Pulmonary Hypertension Network followed in 2016 with a recommendation endorsing the use of iNO in premature infants for the indication of severe pulmonary hypertension [22]. The European Pediatric Pulmonary Vascular Disease Network followed with a similar recommendation in 2019, stating that iNO administration may be considered in preterm infants born <34 weeks of gestation with respiratory failure and confirmed PPHN [23].

### Hypoxic respiratory failure and PPHN in premature infants

There are many causes for HRF in premature infants including parenchymal lung diseases, disorders with increased airway resistance, congenital heart disease (CHD) and PPHN. PPHN results from failure of pulmonary circulatory adaptation at birth. However, in extremely preterm infants born during the canalicular stage of lung development, the reduced cross-sectional area of the pulmonary circulation contributes to the development of PPHN. PPHN is also associated with pulmonary hypoplasia secondary to preterm premature rupture of membrane in this population. PPHN is more prevalent in preterm infants than in term infants [24]. PPHN affects about 8% of extremely premature infants [25]. PPHN in extremely premature infants is an independent risk factor for worse outcomes, including BPD and visual impairment [25–27].

### Studies evaluating iNO in subpopulation of premature infants with PPHN physiology

In preclinical studies, nitric oxide causes a marked increase in pulmonary blood flow in premature ovine fetuses, demonstrating that the pulmonary vasculature is responsive to nitric oxide very early in gestation [28]. Earlier case series suggested the response of premature infants with PPHN physiology to iNO therapy [29,30]. A systematic review evaluating the use of iNO in premature infants with preterm premature rupture of membrane was recently published. This study concluded that iNO improved oxygenation in this population, likely secondary to the high prevalence of PPHN [31]. Two recently published studies evaluated iNO response in premature infants born at <34 weeks of gestation that was initiated for the indication of HRF associated with PPHN. The studies compared the response of iNO in premature infants to the response in term and late preterm infants born at ≥34 weeks of gestation [32,33].

In 2018, Suzuki et al. [32] evaluated the effectiveness of iNO in improving oxygenation in a Japanese cohort of 1106 premature infants with PPHN physiology. The study compared the improvement in oxygenation in 431 iNO-treated infants born at <34 weeks gestational age (GA) with HRF associated with PPHN to 675 iNO-treated infants born at ≥34 weeks GA with the same physiology [32]. The improvement in oxygenation was defined as a 10% decrease from baseline oxygenation index (OI) or a 10% improvement from baseline arterial partial pressure of oxygen (PaO_2_) [34]. In Japan, the use of iNO was approved for term infants, as well as preterm infants with HRF associated with PPHN. The approval was not limited to term and preterm infants born at GA of >34 weeks. As a result, many premature infants received iNO for the indication of HRF associated with PPHN. The diagnosis of PPHN in this study was based on an echocardiogram or clinical judgment and iNO was started within seven days of birth. Limitations of the study include the absence of a prospectively determined outcome. Additionally, congenital diaphragmatic hernia (CDH) was present in 16% of patients in the term/late-preterm group and 1% of the preterm group. Inhaled nitric oxide is known to be less effective in patients with CDH [35].

In 2021, the PaTTerN registry study was published as a non-inferiority study comparing the response to iNO in preterm neonates born at ≥27 to <34 weeks GA versus term and late preterm neonates born at ≥34 to ≤40 weeks GA with HRF associated with PPHN [33]. The study included infants receiving iNO for at least 24 h up to 96 h in the first week of life. The diagnosis of PPHN was based on an echocardiogram or differential oxygen saturations of ≥10% between pre-ductal and post-ductal peripheral oxygen saturation. The primary endpoint was achieving a ≥25% decrease in OI/surrogate oxygenation index (SOI) with iNO treatment. The SOI was based on oxygen saturation when arterial blood gas values were unavailable. A total of 140 neonates were included in the analysis, including 55 infants born at <34 weeks of gestation and 85 infants born at ≥34 weeks of gestation [33].

In both studies, the response rate was similar between the two groups of infants within the study. A comparison of baseline patients’ characteristics and iNO response data is provided in Table 1. The PaTTerN study had a much higher response rate than the Japanese study, although the threshold for a positive response was higher, which may be due to the difference in study design [32–34]. The PaTTerN study included only infants who received iNO for a minimum of 24 h, likely excluding infants who received iNO for a shorter duration of time due to a perceived non-response. The mean time to observe an improvement in oxygenation was much longer than the median in both studies (Table 1), indicating that fewer patients had significantly delayed responses. Most published stewardship programs determine response to iNO after one to two hours of initiation [18,20]. The mean duration of treatment for premature infants in the PaTTerN study was more than twice that of the Japanese study. Both studies are observational studies with inherent limitations including confounding factors such as variations in care between institutions and individual clinicians. Neither of the studies provided data regarding other aspects of cardiorespiratory care of these patients. Specifically, variable ventilatory strategies and the use of other medications for cardiorespiratory support may affect the overall response to iNO [4,36,37]. There was no significant safety concern regarding the use of iNO in these studies.

**Table 1.** Details of response rate, time to response and duration of iNO treatment for patients in two recently published studies.

Parameter	Japanese study	PaTTerN registry study
Age of infants during iNO administration	Initiation of iNO during the first week	Use of iNO during the first week
GA of premature infants, mean (SD)	27.1 (3.5) weeks	29.4 (2.1) weeks
BW of preterm infants, mean (SD)	1043 (593) grams	1324 (481) grams
Overall response rate for term/late preterm	61%	88.2%
Overall response rate for preterm infants	59%	90%
Response time for preterm infants, mean (SD)	9.2 (15.0) hours	10.3 hours
Response time for term/late preterm infants, mean (SD)	12.9 (33.4) hours	15.4 hours
Response time for preterm infants, median	1 hour^a^	5.1 hours
Duration of treatment for term/late preterm infants, mean (SD)	6.0 (12.3) days	6.2 (2.9) days
Duration of treatment for preterm infants, mean	3.5 (5.5) days	8.2 (9.1) days

GA: gestational age; BW: birth weight; iNO: inhaled nitric oxide; SD: standard deviation.

^a^ This is the median response time for all patients; the median response time for preterm infants is not provided separately.

## Conclusions and implications for practice

The debate on the value of using iNO in premature infants born at ≤34 weeks of gestation in the last two decades has evolved. Earlier studies evaluated safety and outcomes such as mortality, prevention of BPD, and other morbidities. Many of these studies included premature infants with HRF and not specifically the subpopulation with PPHN. The most recent studies evaluated the effectiveness of iNO in improving short-term outcomes, namely the improvement in oxygenation in the subset of premature infants with HRF associated with PPHN physiology. These recent studies provide evidence supporting a trial of iNO in premature infants born at GA of ≤34 weeks with HRF associated with PPHN physiology. However, it must be emphasized that prior to initiating iNO in a hypoxic premature infant, clinicians must optimize ventilatory and hemodynamic support. In addition, contraindications to iNO therapy in the patient must be excluded, including CHD with ductal-dependent systemic perfusion, severe left ventricular dysfunction and severe congenital methemoglobinemia. Finally, a diagnosis of PPHN must be made prior to the iNO trial. There are still many challenges and unanswered questions when considering iNO for premature infants. An echocardiogram to diagnose PPHN and exclude CHD may not be readily available for many clinicians [38]. The use of pre- and post-ductal saturation difference may be an alternative option to diagnose PPHN. Once the iNO trial is initiated, it is unclear the duration of therapy before declaring non-response. Long-term effect of iNO in this subpopulation of premature infants with PPHN needs further analysis.

## Data Availability

Data sharing is not applicable to this article as no new data were collected or analysed.
